# Supplementary material for: TLR4 Overexpression Aggravates Bacterial Lipopolysaccharide-Induced Apoptosis via Excessive Autophagy and NF-κB/MAPK Signaling in Transgenic Mammal Models
Source: Cells. 2023 Jul 3;12(13):1769. doi: 10.3390/cells12131769 (PMC10340758; doi:10.3390/cells12131769)
Supplement: Supplementary file 1 [file cells-12-01769-s001.zip › cells-2448556-supplementary.pdf]

Supplemental Information

Table S1. siRNA-specific o*vis-TLR4*

siRNA	Sequence (5’-3’)
Ovis-TLR4-86	*Sense: GCGUACAGGUUGUUCCUAATT*
	Antisense: UUAGGAACAACCUGUACGCTT
Ovis-TLR4-317	*Sense: CCUUGAUACUGACGGGAAATT*
	Antisense: UUUCCCGUCAGUAUCAAGGTT
Ovis-TLR4-1877	*Sense: CCACUUGUCAGAUGAGCAATT*
	Antisense: UUGCUCAUCUGACAAGUGGTT

Table S2. Primers for qRT-PCR analysis

Gene	Accession number	Primer sequence (5’-3’)
TLR4	*NM_001135930.1*	*Forward: CCTTGCGTACAGGTTGTTCCTA*
		Reverse: TTGCTCAATGGTCAGGTTGC
IL-1β	*NM_001009465.2*	*Forward: AGCCGAGAAGTGGTGTTCTG*
		Reverse: TGGCCACCTCTAAAACGTCC
IL-6	*NM_001009392.1*	*Forward: CCTGTCCACTGGGCACATAA*
		Reverse: GTTCAAGCCGCATAGCCATT
TNF-α	*NM_001024860.1*	*Forward: CCAGGCAACTTGCTCTCTCA*
		Reverse: GGCCGATTACCCCGAAGTG
GAPDH	*NM_001190390.1*	*Forward: GTGTCTGTTGTGGATCTGACCTG*
		Reverse: AGAAGAGTGAGTGTCGCTGTTGAAGT

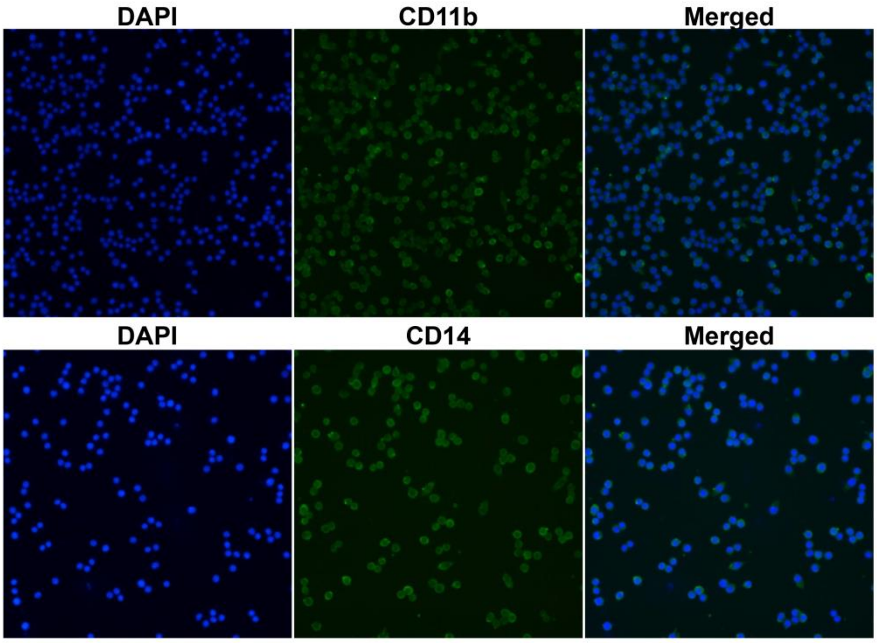

Figure S1. Identification of ovine monocytes/macrophages isolated from peripheral blood. The cellular nuclei were stained by DAPI (Blue), and the expression of monocytes/macrophages -specific proteins (CD14 and CD11b) were detected by immunofluorescence (Green).

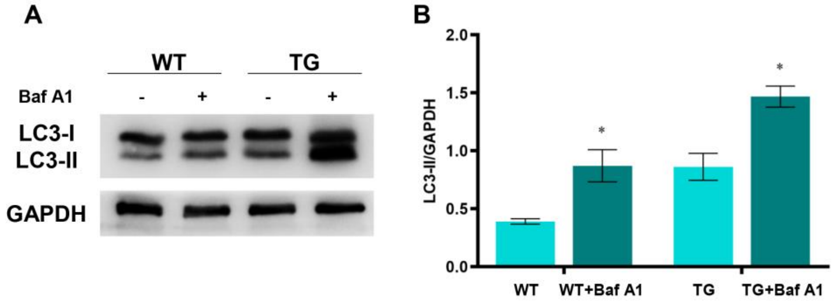

Figure S2. Analysis of the effects of TLR4-overexpression on autophagic flux using bafilomycin A1. Western blotting analysis of LC3 in WT and TG groups treated with or without 10 nM Baf A1. All data are presented as the mean $\pm$ SEM, n$\geqslant$3; *P$<$0.05 vs control group.

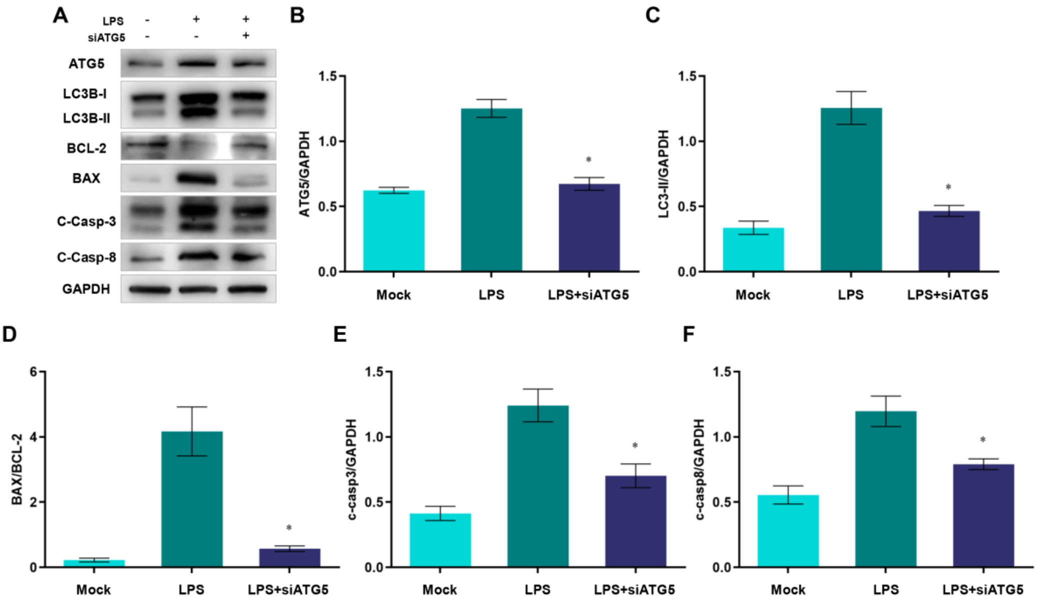

Figure S3. Knocking down of ATG5 reverses the effects of TLR4-overexpression on autophagy and apoptosis. (A) ATG5, LC3B, BCL-2, BAX, cleaved Caspase-3 and cleaved Caspase-8 proteins were examined by Western blotting. GAPDH was used as a loading control. (B-F) ATG5/GAPDH, LC3B-II/GAPDH, BAX/BCL-2, c-Caspase-3/GAPDH and c-Caspase-8/GAPDH ratios of each group based on immunoblotting. All data are presented as the mean ± SEM, n⩾3; *P＜0.05 vs LPS group.
